# Novel Cyclovirus Species in Dogs with Hemorrhagic Gastroenteritis

**DOI:** 10.3390/v13112155

**Published:** 2021-10-26

**Authors:** Kerry Gainor, Yashpal S. Malik, Souvik Ghosh

**Affiliations:** 1 Department of Biomedical Sciences, Ross University School of Veterinary Medicine, Basseterre P.O. Box 334, Saint Kitts and Nevis; KerryGainor@students.rossu.edu; 2 College of Animal Biotechnology, Guru Angad Dev Veterinary and Animal Sciences University, Ludhiana 141001, India; malikyps@gmail.com

**Keywords:** cyclovirus, domestic dogs, complete genome analysis, novel species

## Abstract

Nested PCRs with circovirus/cyclovirus pan-*rep* (replicase gene) primers detected eukaryotic circular Rep-encoding single-stranded DNA (CRESS DNA) viruses in three (samples CN9E, CN16E and CN34) of 18 canine parvovirus-2-positive fecal samples from household dogs with hemorrhagic gastroenteritis on the Caribbean island of Nevis. The complete genomes of CRESS DNA virus CN9E, CN16E and CN34 were determined by inverse nested PCRs. Based on (i) genome organization, (ii) location of the putative origin of replication, (iii) pairwise genome-wide sequence identities, (iv) the presence of conserved motifs in the putative replication-associated protein (Rep) and the arginine-rich region in the amino terminus of the putative capsid protein (Cp) and (v) a phylogenetic analysis, CN9E, CN16E and CN34 were classified as cycloviruses. Canine-associated cycloviruses CN16E and CN34 were closely related to each other and shared low genome-wide nucleotide (59.642–59.704%), deduced Rep (35.018–35.379%) and Cp (26.601%) amino acid sequence identities with CN9E. All the three canine-associated cycloviruses shared < 80% genome-wide pairwise nucleotide sequence identities with cycloviruses from other animals/environmental samples, constituting two novel species (CN9E and CN16E/34) within the genus *Cyclovirus*. Considering the feeding habits of dogs, we could not determine whether the cycloviruses were of dietary origin or infected the host. Interestingly, the CN9E putative Rep-encoding open reading frame was found to use the invertebrate mitochondrial genetic code with an alternative initiation codon (ATA) for translation, corroborating the hypothesis that cycloviruses are actually arthropod-infecting viruses. To our knowledge, this is the first report on the detection and complete genome analysis of cycloviruses from domestic dogs.

## 1. Introduction

Cycloviruses, members of the genus *Cyclovirus* within the family *Circoviridae*, possess a circular, covalently closed, single-stranded DNA genome (~1.7–1.9 kb in length) [1,2]. The cyclovirus genome contains at least two major putative open reading frames (ORFs) that are transcribed bidirectionally [1,2]. The ORF coding for the capsid protein (Cp) is located on the virion-sense (positive-sense) strand, whilst the ORF encoding the replication-associated protein (Rep) is organized on the complementary (antisense) strand of the double-stranded DNA replicative form. Although replication of the cycloviral genome remains to be experimentally investigated, the putative Rep contains conserved sequence motifs that are associated with rolling cycle replication (RCR) [1,2]. The putative Cp is more divergent than Rep and contains an arginine-rich region in the amino terminus that might contribute to DNA-binding activity [1,2,3,4,5]. The 5′-intergenic region (5′-IR, region between the initiation codons of the *rep* and *cp* genes) contains a conserved origin of replication (*ori*), which is marked by the presence of a canonical nonanucleotide motif ‘NAGTATTAC’ (where ‘N’ represents any nucleotide (nt)) located at the apex of a stem–loop structure [1,2,6,7]. The presence of putative introns has been observed within ORFs of various cyclovirus genomes [1,2]. 

Based on genome-wide pairwise nt sequence identities and a phylogenetic analysis, at least 52 cyclovirus species have been formally recognized by the International Committee on Taxonomy of Viruses (ICTV) (https://talk.ictvonline.org/ictv-reports/ictv_online_report/ssdna-viruses/w/circoviridae/660/genus-cyclovirus, accessed on 18 September 2021). Cycloviruses have been detected in various mammals, birds and arthropods [1,2,3,4,5,8,9,10,11,12,13,14,15,16,17,18,19,20,21]. Furthermore, an endogenous cyclovirus element was identified from a rodent-infecting nematode, suggesting that cycloviruses might infect non-arthropod parasitic invertebrates [12]. Since the discovery of cycloviruses was solely based on the identification of viral DNA (by degenerate PCR and metagenomic sequencing) [1,2,3,4,5,8,9,10,11,12,13,14,15,16,17,18,19,20,21], the definitive hosts and pathogenesis of cycloviruses are largely unknown [1,2,4,5,12,13]. However, cycloviruses have been reported in humans with diarrhea [21], encephalitis [21], paralysis [14] and pneumonia [10]. Many cycloviruses have been reported in gut contents/fecal samples, raising speculations that these viruses might be of dietary origin or are actually viruses of enteric parasites [2,5,12]. Based on the wide distribution of cycloviruses in invertebrates (arthropods) and phylogenetic relationships between cyclovirus sequences from vertebrates and arthropods, it has been proposed that cycloviruses are primarily arthropod-infecting viruses [12,13]. 

Within the family *Circoviridae*, members of the genus *Circovirus* have been detected in dogs with vasculitis, hemorrhage, and enteritis [22,23,24,25,26,27] and in foxes with encephalitis [28]. Although the pathogenesis of canine circoviruses remains to be clearly elucidated, the potential role/s of the virus in clinical diseases, directly or as a cofactor, especially with canine parvovirus-2 (CPV-2), has attracted interest [22,23,24,25,26,27]. Based on the analyses of complete genomes, canine circoviruses have been grouped into a single species [1,29]. On the other hand, to date, there have been no reports on the detection and complete genetic makeup of cycloviruses from domestic dogs. We report here the complete genome analysis of cycloviruses from three canine parvovirus-2 (CPV-2)-positive household dogs with hemorrhagic gastroenteritis. 

## 2. Materials and Methods

### 2.1. Ethics Statement

The present study was submitted to the Institutional Animal Care and Use Committee (IACUC) of the Ross University School of Veterinary Medicine (RUSVM), St. Kitts and Nevis. Since the research study was based on leftover samples that were originally collected for diagnostic purposes at the veterinary clinic on the Caribbean island of Nevis, ethical review and approval was waived by the RUSVM IACUC (RUSVM IACUC sample/tissue notification/permission letter number TSU 1.23.21 dated 23 January 2021).

### 2.2. Sampling

During August–October 2020, 39 rectal swabs/fecal samples from household dogs with hemorrhagic gastroenteritis on Nevis Island tested positive for the CPV-2 antigen and/or DNA [30]. Eighteen of the samples were available for the present study, whilst the remaining samples lacked sufficient volumes for further analysis. 

### 2.3. Amplification of Viral DNA

The extraction of viral DNA was performed using the QIAamp Fast DNA Stool Mini Kit (Qiagen Sciences, Germantown, MD, USA) according to the manufacturer’s instructions. The samples were screened for the presence of eukaryotic circular Rep-encoding single-stranded DNA (CRESS DNA) viruses by nested PCR assays using pan-*rep* primers (primers CV-F1, CV-R1, CV-F2 and CV-R2) that target a short stretch (~400 bp) of the *rep* gene, as described by Li et al. [3]. The complete genomes of the canine-associated cycloviruses were amplified by inverse nested PCRs using additional primers that were designed from the partial *rep* sequences (Supplementary Table S1). PCRs were carried out using Platinum™ Taq DNA Polymerase (Invitrogen™, Thermo Fisher Scientific Corporation, Waltham, MA, USA) following the manufacturer’s instructions. The negative control consisted of sterile water in all PCR reactions. 

### 2.4. Nucleotide Sequencing

The PCR products were purified using the Wizard^®^ SV Gel and PCR Clean-Up kit (Promega, Madison, WI, USA) according to manufacturers’ instructions. Nucleotide sequences were determined using the ABI Prism Big Dye Terminator Cycle Sequencing Ready Reaction Kit (Applied Biosystems, Foster City, CA, USA) on an ABI 3730XL Genetic Analyzer (Applied Biosystems, Foster City, CA, USA).

### 2.5. Sequence Analysis

The maps of the cyclovirus genomes were created with the ‘Draw Custom Plasmid Map’ program (https://www.rf-cloning.org/savvy.php, accessed on 8 September 2021). The putative stem–loop structure was located on the viral genome using the mFold program [31]. Putative ORFs coding for the viral Rep and Cp were determined using the ORF finder (https://www.ncbi.nlm.nih.gov/orffinder/, accessed on 10 September 2021). On the other hand, Rep coding sequences separated by a putative intron were identified using the ExPASy translate tool (https://web.expasy.org/translate/, accessed on 10 September 2021) and BLASTX program (Basic Local Alignment Search Tool, www.ncbi.nlm.nih.gov/ blast, accessed on 11 September 2021). The standard BLASTN and BLASTP programs were employed to conduct a homology search for the related nt and deduced amino acid (aa) sequences, respectively. Pairwise sequence (%) identities for the complete viral genomes and the putative Rep and Cp were calculated using the MUSCLE algorithm embedded in the SDTv1.2 program, as described in previous studies [2,32]. 

The complete genomes of canine-associated cycloviruses were examined for recombination events using the RDP4 program with default parameters [33]. A cyclovirus sequence was considered as a recombinant if it was supported by two, or more than two, detection methods (3Seq, BOOTSCAN, CHIMAERA, GENECONV, MAXCHI, RDP and SISCAN) with a highest acceptable *p*-value of *p* < 0.01 with Bonferroni’s correction [15,33]. Multiple alignments of the cyclovirus sequences were performed with the MUSCLE algorithm in MEGA7 software [34]. A phylogenetic analysis was carried out by the maximum likelihood (ML) method using MEGA7 software [34] with the GTR+G model of substitution and 1000 bootstrap replicates, as described in previous studies [2,15]. 

### 2.6. GenBank Accession Numbers

The GenBank accession number for the complete genome sequence of canine-associated cycloviruses CN9E, CN16E and CN34 are OK148727, OK148728 and OK148729, respectively. 

## 3. Results and Discussion

In the present study, nested PCRs with circovirus/cyclovirus pan-*rep* primers generated the expected ~400-bp amplicon in three (samples CN9E, CN16E and CN34) of the 18 CPV-2 (new CPV-2a)-positive fecal samples from household dogs with hemorrhagic gastroenteritis on Nevis Island. All three PCR-positive dogs were Island mix. Island mix is a cross between a local canine breed native to St. Kitts and another canine breed imported to the island, especially from the North Americas. Most Island mix dogs are medium-built, with a foxlike face, large upright ears, and a crooked tail. Dog CN16E (aged 9 weeks during sampling) died, whilst CN9E and CN34 (aged 4 months and 7 months, respectively) eventually recovered. The presence of CRESS DNA viruses was confirmed by sequencing the partial rep gene and BLASTN analysis. 

**Figure 1:**
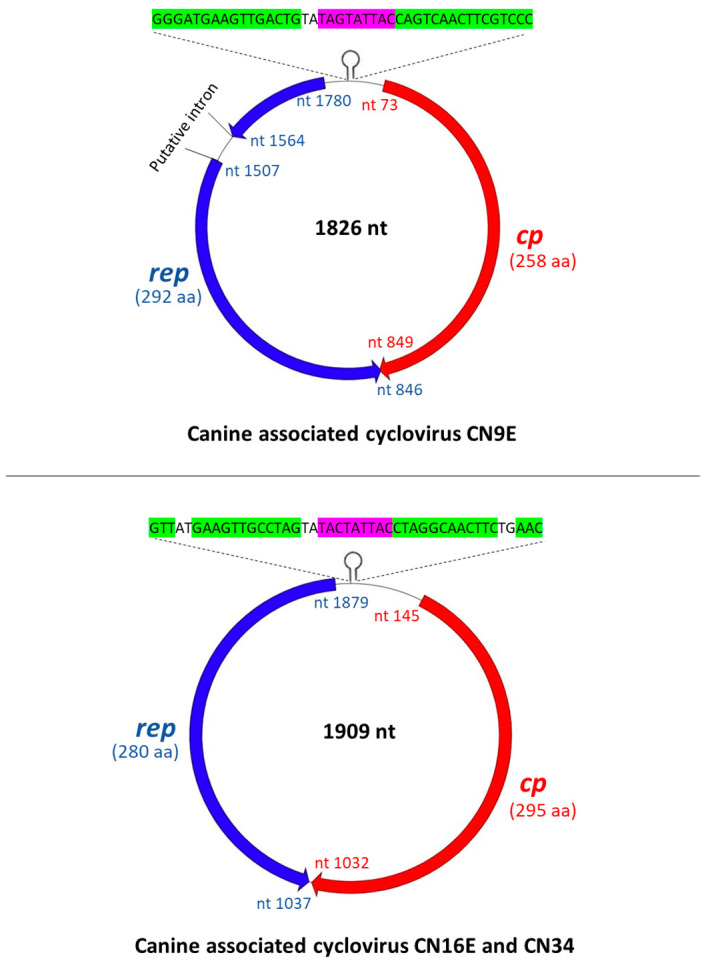
The organization of the complete genomes of canine associated cycloviruses CN9E, CN16E and CN34. The inversely arranged open reading frames coding for the putative replication-associated (Rep) and capsid (Cp) proteins are shown with blue and red arrows, respectively. The putative origin of replication (ori), marked by a nonanucleotide motif (highlighted with pink) at the apex of the stem–loop structure (complementary regions forming the ‘putative stem’ are shown with green), is shown in the 5′-intergenic region. Following the recommendations of the International Committee on Taxonomy of Viruses [1,2], the first nucleotide (nt) of the nonanucleotide motif was recognized as nt ‘position one’ of the cyclovirus genomes. The sizes of Rep and Cp are mentioned in parentheses. nt: nucleotide; aa: amino acid.

**Figure 2:**
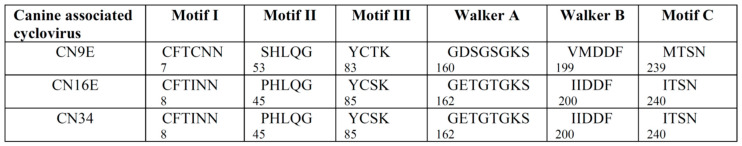
The conserved rolling circle replication (motifs I–III) and superfamily 3 helicase (Walkers A and B and motif C) motifs were retained in the putative replication-associated proteins (Rep) of the canine-associated cycloviruses. The number below the motif sequence corresponds to the position of the amino acid residue in the putative Rep protein.

**Figure 3:**
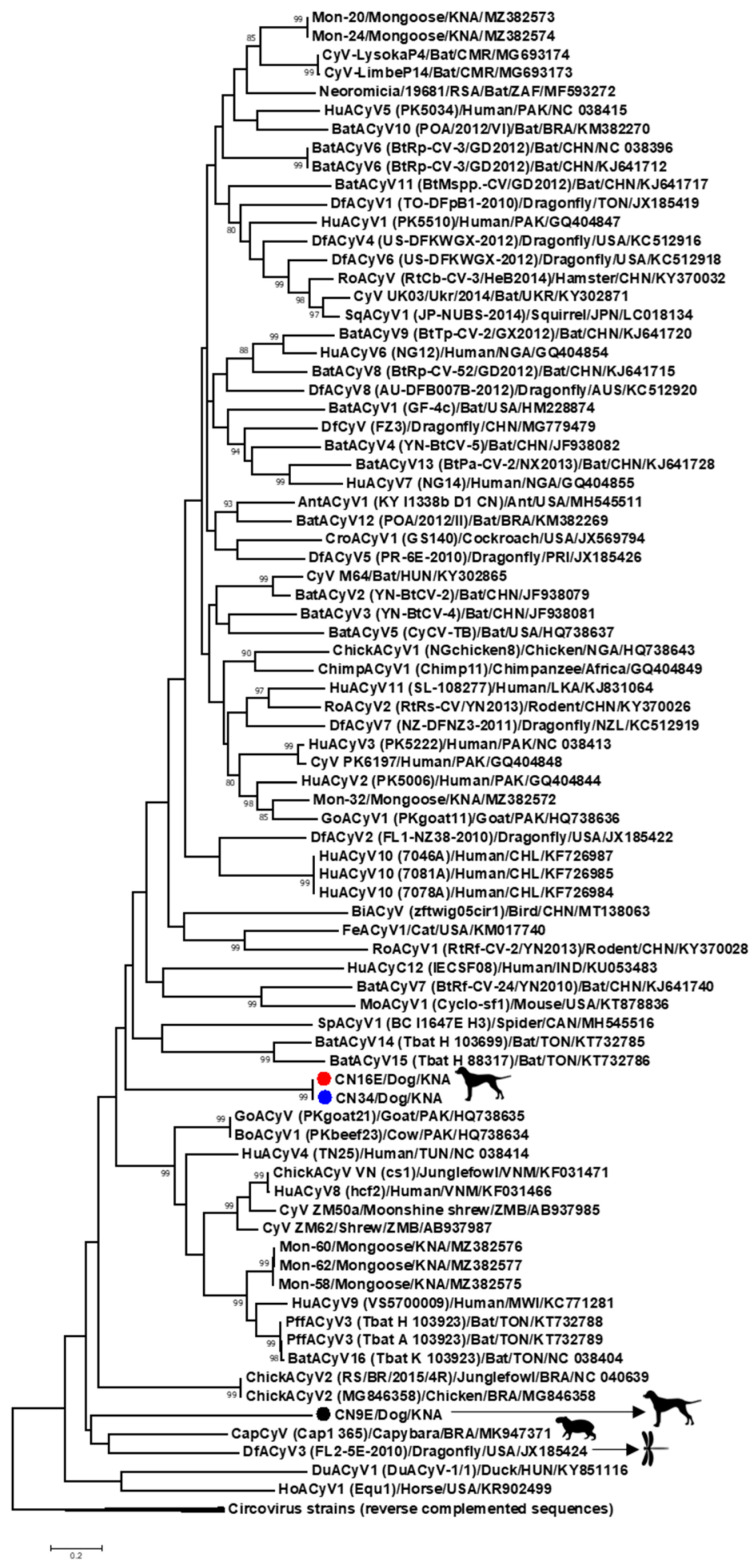
Phylogenetic analysis based on the complete genome sequences of canine-associated cycloviruses CN9E, CN16E and CN34 with those of other cycloviruses. The virus name/source (detected in the animal species)/country are mentioned for the ‘CN’ sequences, while the species or virus name (isolate)/source (detected in the animal species)/country/GenBank accession number are shown for the other cyclovirus sequences. Following the recommendations of the International Committee on Taxonomy of Viruses [1,2], the first nucleotide (nt) of the nonanucleotide motif was recognized as nt ‘position one’ of the cyclovirus genomes. The tree was rooted out with circovirus reverse complement sequences as the outgroup. Scale bar, 0.2 substitutions per nucleotide. Bootstrap values of < 80 are not shown. CN9E, CN16E and CN34 are shown with a black, red and a blue circle, respectively. AntACyV: ant-associated cyclovirus; BatACyV: bat-associated cyclovirus; BiACyV: bird-associated cyclovirus; BoACyV: bovine-associated cyclovirus; CapyACyV: capybara-associated cyclovirus; ChickACyV: chicken-associated cyclovirus; ChimpACyV: chimpanzee-associated cyclovirus; CroACyV: cockroach-associated cyclovirus; CyV: cyclovirus; DfACyV: dragonfly-associated cyclovirus; DuACyV: duck-associated cyclovirus; FeACyV: feline-associated cyclovirus; GoACyV: goat-associated cyclovirus; HoACyV: horse-associated cyclovirus; HuACyV: human-associated cyclovirus; MoACyV: mouse-associated cyclovirus; PffACyV: Pacific flying fox-associated cyclovirus; RoACyV: rodent-associated cyclovirus; SpACyV: spider-associated cyclovirus; SqACyV: squirrel-associated cyclovirus.

Since the classification of CRESS DNA viruses into circoviruses/cycloviruses is based on genome-wide analyses [1,2], the complete genome sequences of CN9E, CN16E and CN34 were determined by inverse nested PCRs using primers designed from the partial rep sequences of their respective virus strains (Supplementary Table S1). Based on (i) genome organization, (ii) the location of the putative *ori* (present on the Cp-encoding strand), (iii) pairwise genome-wide sequence identities (>55% nt sequence identities with other cycloviruses), (iv) the presence of conserved RCR and superfamily 3 helicase motifs in Rep and the arginine-rich region in the amino terminus of Cp and (v) a phylogenetic analysis, CN9E, CN16E and CN34 were classified as cycloviruses (Figure 1, Figure 2 and Figure 3, Table 1 and Supplementary Figures S2–S5). Following the recommendations of ICTV [1,2], the first nt of the nonanucleotide motif was recognized as nt ‘position one’ of the canine-associated cyclovirus genomes. Since recombinants have been detected within the genus *Cyclovirus* [16,35], the canine-associated cycloviruses were examined for potential recombination events. Only CN9E appeared to be a potential recombinant with dragonfly-associated cyclovirus 4 isolate US-DFKWGX-2012 [35] as the minor parent and an unknown major parent (Supplementary Figure S6). 

### 3.1. Complete Genome Analysis of Canine-Associated Cyclovirus CN9E 

The complete genome of canine-associated cyclovirus CN9E was 1826 nt in length, which was comparable to those of other cycloviruses (Figure 1) [1,2]. The putative *ori*, characterized by the presence of a canonical nonanucleotide motif ‘TAGTATTAC’ at the apex of a potential stem–loop structure, was located on the 5′-IR region (Figure 1). The 3’-IR was absent (Figure 1). Based on genome-wide pairwise nt sequence identities, CN9E shared a maximum identity of 61.646% with human-associated cyclovirus 8 isolate hcf2 (detected in the cerebrospinal fluid of a human with acute central nervous system infection in Vietnam) [36], followed by an identity of 61.337% with capybara-associated cyclovirus 1 isolate Cap1_365 from fecal pellets of a capybara in Brazil [37] (Table 1 and Supplementary Figure S3). Pairwise identities of 59.642% and 59.704% were observed with canine-associated cycloviruses CN34 and CN16E, respectively (Table 1). Phylogenetically, the complete genome sequence of CN9E formed a distinct branch near the cluster of capybara-associated cyclovirus 1 isolate Cap1_365 [37] and dragonfly cyclovirus 3 isolate FL2-5E-2010 from the USA [38] within the clade of cycloviruses (Figure 3). According to the ICTV, cycloviruses sharing <80% genome-wide pairwise sequence identities with members of known species are assigned to new species [1,2]. Based on the ICTV guidelines, canine-associated cyclovirus CN9E qualifies as a new species within the genus *Cyclovirus*.

Using the standard genetic code (transl_table = 1) (https://www.ncbi.nlm.nih.gov/ Taxonomy/Utils/wprintgc.cgi, accessed 11 September 2021) with ‘ATG’ as the initiation codon, the CN9E sequence was found to lack a putative Rep-encoding ORF. Since alternative initiation codons have been proposed for a number of avian circoviruses [3], we repeated the analysis using the standard genetic code with alternative initiation codons, revealing a putative ORF (nt 1541–nt 846) that encoded a 231-aa polypeptide (starting with aa residue methionine, encoded by ‘TTG’). Although the putative polypeptide shared a maximum homology with the cyclovirus Rep sequences, it lacked the Rep amino terminal region, including the presence of the conserved RCR motifs I and II (Supplementary Figure S7). Interestingly, using the invertebrate mitochondrial genetic code (transl_table = 5) with an alternative initiation codon (ATA), we identified putative Rep coding sequences that were separated by a putative intron (nt 1563–nt 1508) with a canonical splice donor site (GT) and splice acceptor site (AG) (Figure 1 and Supplementary Figure S7). The resultant CN9E putative Rep (292 aa in size) retained all the conserved RCR and superfamily 3 helicase motifs that are characteristic of cycloviruses (Figure 2 and Supplementary Figure S7) and shared a maximum pairwise deduced aa identity of 47.272% with dragonfly cyclovirus 3 isolate FL2-5E-2010 from the USA [38] (Table 1 and Supplementary Figure S4). By a phylogenetic analysis, the CN9E Rep formed an isolated branch within a cluster that consisted of capybara-associated cyclovirus 1 isolate Cap1_365 (deduced aa identity of 44.086% with CN9E) [37] and dragonfly cyclovirus 3 isolate FL2-5E-2010 [38] (Supplementary Figure S5). 

Although the CN9E genome encoded a putative capsid protein of the same size (258 aa) using both the standard genetic code and invertebrate mitochondrial code, 7 aa mismatches were observed between the putative Cps derived using their respective genetic codes (Supplementary Figure S8). Since the CN9E genome used the invertebrate mitochondrial genetic code for translation of the putative Rep, to maintain the consistency, we used the CN9E putative Cp generated using the invertebrate mitochondrial genetic code for further analysis. The CN9E putative Cp shared a maximum deduced aa identity of 38.201% with CRESS DNA virus clone CG130 from wastewater in the USA (GenBank accession number KY487801) (Table 1).

It is intriguing that the canine-associated cyclovirus CN9E genome contained a putative Rep-encoding ORF that used the invertebrate mitochondrial genetic code (with the alternative initiation codon ‘ATA’) for translation. To date, only a few viruses have been shown/proposed to infect the mitochondria. Notable among these are mitoviruses (genus *Mitovirus*, family *Narnaviridae*) [39,40]. Mitoviruses are plus-stranded RNA virus-like elements that lack a capsid and replicate in the mitochondria of fungi [39,40]. Recently, unique picobirnavirus-like (double-stranded RNA viruses that belong to the family *Picobirnaviridae*) sequences that use an alternative mitochondrial genetic code (invertebrate mitochondrial code, transl_table = 5, or mold mitochondrial genetic code, transl_table = 4) for translation of the putative RNA-dependent RNA polymerase have been reported in bats, humans, invertebrates (crustaceans and myriapods) and a mongoose [18,41,42,43]. Among DNA viruses, there is evidence suggesting that damselfish virus-like agents replicate in the mitochondria of host cells [44]. Based on the wide distribution and diversity of cycloviruses and other CRESS DNA viruses in invertebrates, and the random interspersion of endogenous circoviral elements from insect genomes with cyclovirus sequences from vertebrate samples, it has been proposed that cycloviruses actually infect invertebrates and that cyclovirus sequences from vertebrate samples reflect their widespread presence in the environment as viruses of parasitic arthropods [12,13]. The identification of a cyclovirus sequence containing a putative Rep-encoding ORF that uses the invertebrate mitochondrial genetic code for translation corroborates this hypothesis. However, the present observation was based on sequence analysis and warrants further investigation.

### 3.2. Complete Genome Analysis of Canine-Associated Cyclovirus CN16E and CN34

The complete genomes of canine-associated cycloviruses CN16E and CN34 were 1909 nt in length, which was larger than those of most cycloviruses (Figure 1). CN16E and CN34 shared an identical genome organization. Although the putative *ori* was retained in the 5′-IR, the nonanucleotide motif ‘TACTATTAC’ atop the stem–loop structure differed in a single nt compared to that (TAGTATTAC) observed in most cycloviruses [1,2] (Figure 1). The 3’-IR extended from nt 1033 to nt 1036 (Figure 1). The complete genomes of CN16E and CN34 shared a nt sequence identity of 99.790% between them (Table 1). With other cycloviruses, CN16E and CN34 shared maximum genome-wide pairwise identities of 63.010% and 63.040%, respectively, with feline-associated cyclovirus 1 from the fecal sample of an apparently healthy cat in the USA [45] (Table 1 and Supplementary Figure S3). Phylogenetically, the complete genome sequences of CN16E and CN34 formed an isolated cluster within the clade of cycloviruses (Figure 3). Based on the ICTV classification system [1,2], CN16E and CN34 were assigned to a novel species within the genus *Cyclovirus*. 

Putative Rep- and Cp-encoding ORF of CN16E and CN34 used the standard genetic code (transl_table = 1) for translation. The CN16E and CN34 Rep (280 aa in size) contained the conserved RCR and superfamily 3 helicase motifs (Figure 2) and shared deduced aa identities of 99.285% between them and < 50% pairwise identities with Rep of other cycloviruses (Table 1 and Supplementary Figure S4). By a phylogenetic analysis, the CN16E and CN34 Rep branched into an isolated group near the cluster consisting of CN9E, capybara-associated cyclovirus 1 isolate Cap1_365 [37] and dragonfly cyclovirus 3 isolate FL2-5E-2010 [38] (Supplementary Figure S5). The CN16E and CN34 putative Cp (295 aa in size) shared absolute sequence identities between them (Table 1). With other viruses, maximum/significant pairwise deduced aa identities of 30.401% were observed with CRESS DNA virus clone CG130 and 28.601% with chicken-associated cyclovirus 2 strain RS/BR/2015/4R (Table 1). 

Although there is a lack of conclusive data on the definitive hosts and pathogenic potential of cycloviruses [1,2,4,5,12,13], the detection of novel cyclovirus species in CPV-2-positive dogs with hemorrhagic gastroenteritis might be of interest, necessitating further studies on the prevalence of cycloviruses in diarrheic dogs, especially those infected with CPV-2. Members of the family *Circoviridae*, including canine circoviruses, have been associated with clinical conditions and immunosuppression [1,2,4,5,22,23,24,25,26,27,46,47]. Recent studies have proposed a synergistic effect of canine circovirus and CPV-2 coinfections attributing to the development of acute clinical disease in dogs, even in animals that are vaccinated against CPV-2 [24,25,46,47]. Based on these observations, the possible role/s of cycloviruses in CPV-2-infected dogs warrant further investigation. On the other hand, since the present study was based on fecal, and not tissue, samples, we could not establish whether the canine-associated cycloviruses caused infections in the host. Dogs are polyphagous omnivores that are known to feed on invertebrates, including arthropods. It might be possible that the canine-associated cycloviruses actually originated from consumed food, such as invertebrates (arthropods). The invertebrate origins of the canine-associated cycloviruses viruses were supported by the detection of cyclovirus CN9E that used an invertebrate mitochondrial genetic code to translate the putative Rep. 

## 4. Conclusions

To our knowledge, the present study is the first report on the detection and complete genome analysis of cycloviruses from CPV-2-positive domestic dogs with hemorrhagic gastroenteritis. Since our study was based on cyclovirus sequences from fecal samples, we could not determine if these viruses actually infected the dogs or were of dietary origin, possibly from the consumption of invertebrates (arthropods). This observation was corroborated by the identification of a cyclovirus sequence containing a putative Rep-encoding ORF that used the invertebrate mitochondrial genetic code (with alternative initiation codon ‘ATA’) for translation, supporting the hypothesis that cycloviruses are mainly arthropod-infecting viruses [12,13]. Following the ICTV classification system [1,2], two novel species were recognized within the genus *Cyclovirus*. Future studies based on screening for antibodies against cycloviruses, the identification of cyclovirus DNA in tissue samples, the propagation of cycloviruses in cell lines and inoculations in gnotobiotic animals alongside extensive surveillance and the complete genome analyses of cycloviruses in various invertebrates are required to decipher the true host/s and pathogenic potentials of members of the genus *Cyclovirus*. 

## Figures and Tables

**Table 1:** Maximum/significant pairwise sequence (%) identities of the complete genomes, putative replication-associated proteins (Rep) and putative capsid proteins (Cp) of canine-associated cycloviruses from domestic dogs between themselves and with those from other animal species or environmental samples.

		Canine-Associated Cyclovirus (*GenBank Accession Number*)		
		CN9E (*OK148727*)	CN16E (*OK148728*)	CN34 (*OK148729*)
Maximum/significant pairwise nucleotide sequence (%) identities of complete genome	Between canine-associated cycloviruses	59.704% with CN16E 59.642% with CN34	99.790% between CN16E and CN34	
	With cyclovirus (Strain name/Detected in animal species/Country/Year/GenBank accession number) from other animal species	61.646% with human-associated cyclovirus 8 isolate hcf2/Human/ Vietnam/2009/KF031466 61.337% with capybara-associated cyclovirus 1 isolate Cap1_365/ Capybara/Brazil/2016/MK947371	63.010% with feline-associated cyclovirus 1/Cat/USA/2013/ KM017740 62.910% with dragonfly-associated cyclovirus 1 isolate TODFpB12010/ Dragonfly/Tonga/2010/JX185419	63.040% with feline-associated cyclovirus 1/Cat/USA/2013/ KM017740 62.709% with dragonfly-associated cyclovirus 1 isolate TODFpB12010/ Dragonfly/Tonga/2010/JX185419
Maximum/significant pairwise deduced amino acid (aa) sequence (%) identities of putative Rep	Between canine-associated cycloviruses	35.379% with CN34 35.018% with CN16E	99.285% between CN16E and CN34	
	With cyclovirus (Strain name/Detected in animal species/Country/Year/GenBank accession number) from other animal species	47.272% with dragonfly cyclovirus 3 isolate FL2-5E-2010/Dragonfly/ USA/2010/JX185424 45.112% with human-associated cyclovirus 5 isolate PK5034/ Human/Pakistan/2007/NC_038415	49.462% with capybara-associated cyclovirus 1 isolate Cap1_365/ Capybara/Brazil/2016/MK947371 49.446% with dragonfly-associated cyclovirus isolate DfCyV-FZ3/ Dragonfly/China/2016/MG779479	49.820% with capybara-associated cyclovirus 1 isolate Cap1_365/ Capybara/Brazil/2016/MK947371 49.815% with dragonfly-associated cyclovirus isolate DfCyV-FZ3/ Dragonfly/China/2016/ MG779479
Maximum/significant pairwise deduced aa sequence (%) identities of putative Cp	Between canine-associated cycloviruses	26.601% with CN16E and CN34	100% between CN16E and CN34	
	With cyclovirus/CRESS DNA virus (Strain name/Detected in animal species, or environmental sample/Country/Year/GenBank accession number) from other animal species/environmental sample	38.201% with uncultured virus clone CG130/Wastewater/USA/ 2015/ KY487801 30.901% with cyclovirus strain ZM41/Red musk shrew/Zambia/ 2012/AB937984	30.401% with uncultured virus, clone CG130/Wastewater/USA/2015/ KY487801 28.601% with chicken-associated cyclovirus 2 strain RS/BR/2015/4R/ Brazil/2015/NC_040639	

## Data Availability

Not applicable.

## Supplementary Materials

The following are available online at https://www.mdpi.com/article/10.3390/v13112155/s1: Supplementary Table S1: Additional primers employed in inverse nested PCRs to amplify the complete genomes of canine-associated cycloviruses. Supplementary Figure S2: The conserved arginine-rich region was retained in the amino terminus of the putative capsid proteins (Cp) of canine-associated cycloviruses CN9E, CN16E and CN34. Supplementary Figure S3: Two-dimensional graphical representation of the pairwise nucleotide sequence (%) identities between the complete genomes of the cycloviruses. Supplementary Figure S4: Two-dimensional graphical representation of the pairwise deduced amino acid sequence (%) identities between the putative Rep of the cycloviruses. Supplementary Figure S5: Phylogenetic analysis of the putative replication-associated proteins (Rep) of canine-associated cycloviruses CN9E, CN16E and CN34 with those of other cycloviruses. Supplementary Figure S6: Identification of a putative recombination event involving canine-associated cyclovirus CN9E and dragonfly-associated cyclovirus 4 isolate US-DFKWGX-2012/Dragonfly/USA/KC512916, as determined by the Recombination Detection Program (RDP v.4.101). Supplementary Figure S7: The putative Rep-encoding open reading frame of canine-associated cyclovirus CN9E. Supplementary Figure S8: Alignment of the CN9E putative deduced capsid (Cp) amino acid (aa) sequences derived using the standard genetic code (CN9E/Cp/STD_code) with ‘ATG’ as the initiation codon and invertebrate mitochondrial genetic code (CN9E/Cp/INV-Mit_code) with ‘ATG’ as the initiation codon.
