# Supplementary material for: Novel Cyclovirus Species in Dogs with Hemorrhagic Gastroenteritis
Source: Viruses. 2021 Oct 26;13(11):2155. doi: 10.3390/v13112155 (PMC8622408; doi:10.3390/v13112155)
Supplement: Supplementary file 1 [file viruses-13-02155-s001.zip › Supplementary figure S2.pdf]

Supplementary figure S2. The conserved arginine rich region was retained in the amino terminus of the putative capsid proteins (Cp) of canine associated cyclovirus CN9E, CN16E and CN34. A '-' denotes absence of an amino acid (aa) residue at that position. Numbers to the right indicate the positions of the aa for respective sequences.

```
CN9E_Cp         -----MRRRISRFRRRYRLRTQSRYRKRVKTSSLVRHHRALSSTDN---LTVYLTARVRL 52
CN16E_Cp        MYRRTFRRRFRRYRRRFRRFSRFRNRRFARAHKTRRHLASGGQYFTRLVRSETWDTSQSY 60
CN34_Cp         MYRRTFRRRFRRYRRRFRRFSRFRNRRFARAHKTRRHLASGGQYFTRLVRSETWDTSQSY 60
```
