# Supplementary material for: Novel Cyclovirus Species in Dogs with Hemorrhagic Gastroenteritis
Source: Viruses. 2021 Oct 26;13(11):2155. doi: 10.3390/v13112155 (PMC8622408; doi:10.3390/v13112155)

**Supplementary figure S4.** Two-dimensional graphical representation of pairwise deduced amino acid sequence (%) identities between the putative Rep of cycloviruses. The plot was created using the Sequence Demarcation Tool Version 1.2 (SDTv1.2) with the MUSCLE alignment algorithm, as described previously [2]. The canine associated cycloviruses are highlighted with asterisks ‘*’.

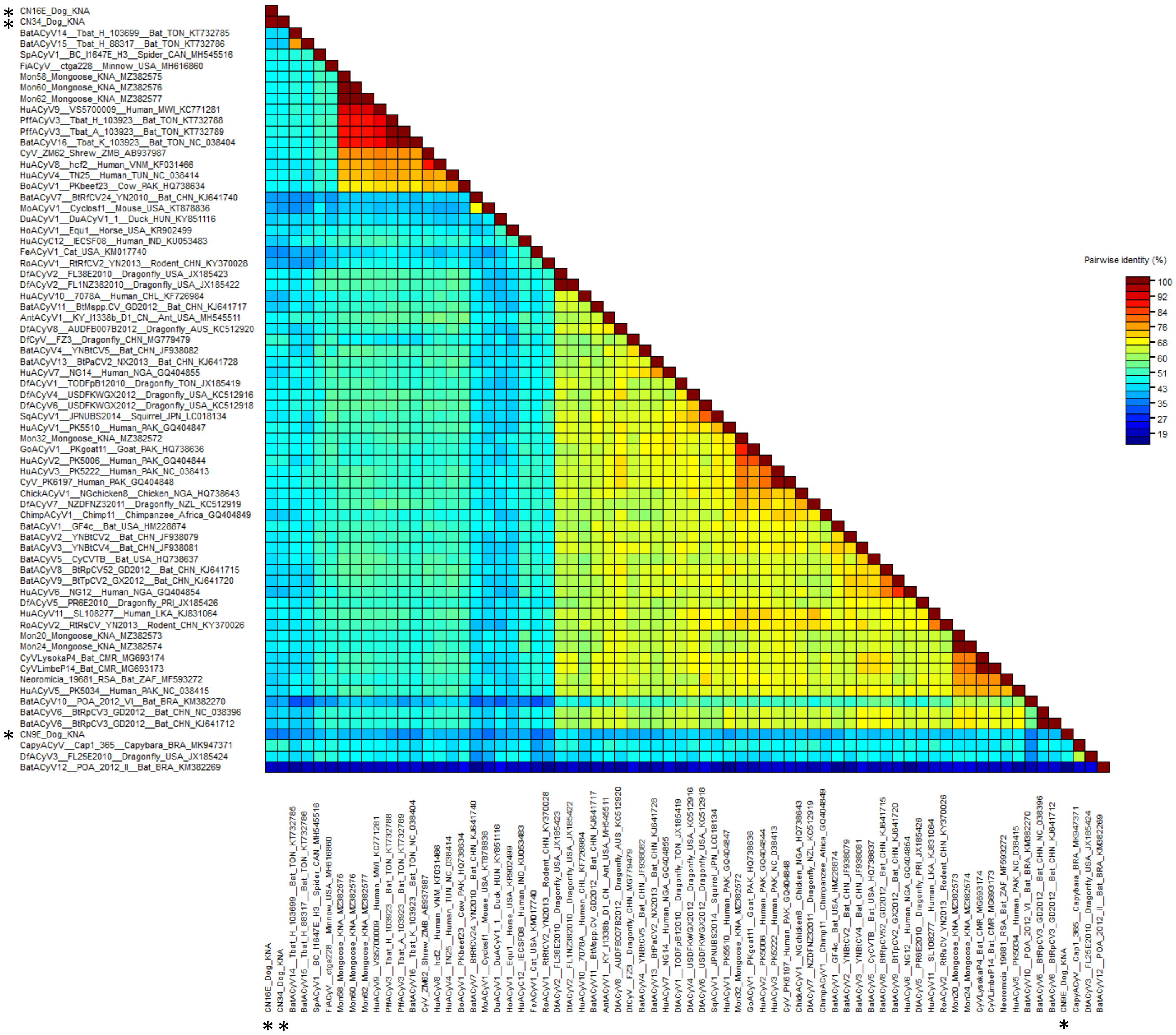

Supplement: Supplementary file 1 [file viruses-13-02155-s001.zip › Supplementary figure S4.pdf]
