# Supplementary material for: Novel Cyclovirus Species in Dogs with Hemorrhagic Gastroenteritis
Source: Viruses. 2021 Oct 26;13(11):2155. doi: 10.3390/v13112155 (PMC8622408; doi:10.3390/v13112155)

Supplementary figure S5. Phylogenetic analysis of the putative replication-associated proteins (Rep) of canine associated cyclovirus CN9E, CN16E and CN34 with those of other cycloviruses. The virus name/source (detected in animal species)/country are shown for the 'CN' sequences, while the species, or virus name (isolate)/source (detected in animal species)/country/GenBank accession number have been mentioned for the other cyclovirus sequences. The tree was created by the maximum likelihood (ML) method, with the LG model of substitution and 1000 bootstrap replicates. Circovirus putative Rep sequences constituted the outgroup. Scale bar, 2 substitutions per amino acid. Bootstrap values of < 80 are not shown. CN9E, CN16E, and CN34 is shown with a black, red, and blue circle, respectively. AntACyV: ant associated cylcovirus; BatACyV: bat associated cyclovirus; BoACyV: bovine associated cyclovirus; CapyACyV: capybara associated cyclovirus; ChickACyV: chicken associated cyclovirus; ChimpACyV: chimpanzee associated cyclovirus; CroACyV: cockroach associated cyclovirus; CyV: cyclovirus; DfACyV: dragonfly associated cycovirus; DuACyV: duck associated cyclovirus; FeACyV: feline associated cyclovirus; FiACyV: Fish associated cyclovirus; GoACyV: goat associated cyclovirus; HoACyV: horse associated cyclovirus; HuACyV: human associated cyclovirus; MoACyV: mouse associated cyclovirus; PffACyV: Pacific flying fox associated cyclovirus; RoACyV: rodent associated cyclovirus; SpACyV: spider associated cyclovirus; SqACyV: squirrel associated cyclovirus.

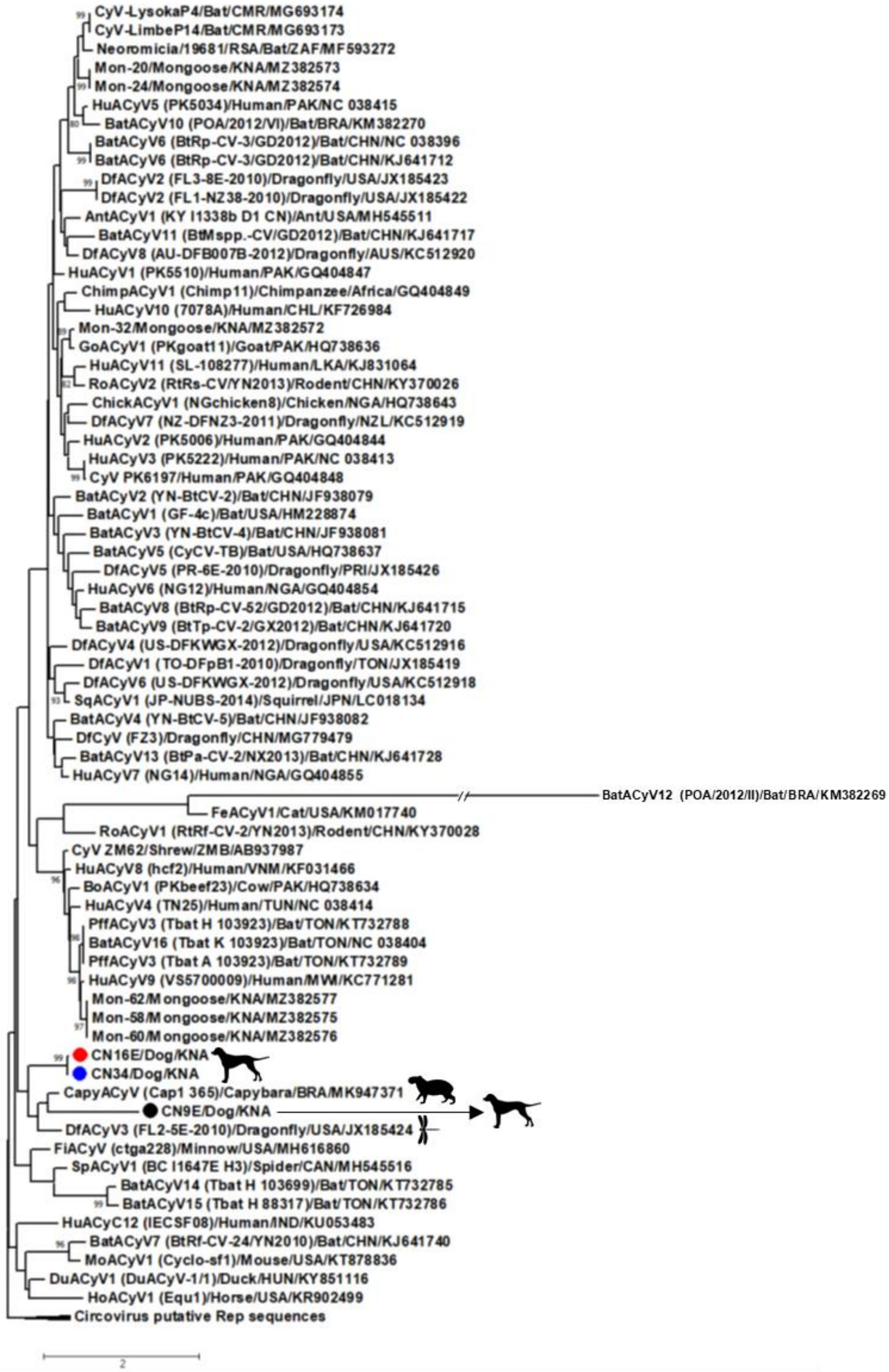

Supplement: Supplementary file 1 [file viruses-13-02155-s001.zip › Supplementary figure S5.pdf]
