# Supplementary material for: Novel Cyclovirus Species in Dogs with Hemorrhagic Gastroenteritis
Source: Viruses. 2021 Oct 26;13(11):2155. doi: 10.3390/v13112155 (PMC8622408; doi:10.3390/v13112155)
Supplement: Supplementary file 1 [file viruses-13-02155-s001.zip › Supplementary figure S6.pdf]

Supplementary figure S6. Identification of putative recombination event involving canine associated cyclovirus CN9E and dragonfly associated cyclovirus 4 isolate US-DFKWGX-2012/Dragonfly/USA/KC512916, as determined by the Recombination Detection Program (RDP v.4.101). A sequence was considered as a recombinant if it was identified by two or more than two methods with a highest acceptable *p-value* of $p < 0.01$ with Bonferroni's correction.

Recombinant: Canine associated cyclovirus CN9E/Dog/KNA
Major parent: Unknown
Minor parent: Dragonfly associated cyclovirus 4 isolate US-DFKWGX-2012/Dragonfly/USA/KC512916
Beginning breakpoint: nt 1303 (without gaps)
Ending breakpoint: nt 1481 (without gaps)

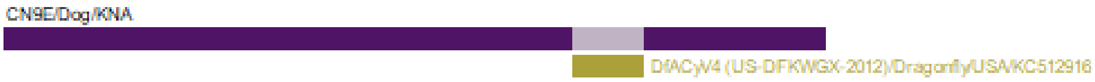

Confirmation table generated by RDP v.4.101 program.

Method	***Average p-value***
RDP	4.534×10^{-02}
MaxChi	2.027×10^{-02}
Chimaera	1.384×10^{-02}
