# Supplementary material for: Novel Cyclovirus Species in Dogs with Hemorrhagic Gastroenteritis
Source: Viruses. 2021 Oct 26;13(11):2155. doi: 10.3390/v13112155 (PMC8622408; doi:10.3390/v13112155)
Supplement: Supplementary file 1 [file viruses-13-02155-s001.zip › Supplementary figure S7.pdf]

Supplementary figure S7 (A and B). The putative Rep encoding open reading frame of canine associated cyclovirus CN9E.

(A) The reverse complement nucleotide sequence of canine associated cyclovirus CN9E showing the putative Rep ORF (highlighted with green) and the corresponding putative deduced Rep amino acid sequence (highlighted with blue). CN9E contained a putative Rep encoding ORF (highlighted with green) that used the invertebrate mitochondrial genetic code (transl_table=5) with alternative initiation codon 'ATA' (shown with purple font) for translation. The putative Rep coding sequences (nt 1780-nt 1564 and nt 1507-nt 846) was separated by a putative intron (nt 1563-nt 1508) (highlighted with yellow) with a canonical splice donor site (GT) and splice acceptor site (AG) (shown with red font). The conserved rolling cycle replication and superfamily 3 helicase motifs in the putative Rep sequence are underlined. The stop codon is shown with brown font and asterisks '*'. The analysis was performed using the ExPASy translate tool (https://web.expasy.org/translate/, accessed on September 10, 2021) and BLASTX program (Basic Local Alignment Search Tool, www.ncbi.nlm.nih.gov/blast, accessed on September 11, 2021).

```
1826                                                         1780 ------->
tacagtcaacttcatcccatcgattgtggcgaactctggtggtcgaatacgtggtgtgcgg
                                                M  R  G  V  R
tggtgtttcacttgcaacaactatacagaagaggatgagcgtgccgtccgagctctcact
 W  C  F  T  C  N  N  Y  T  E  E  D  E  R  A  V  R  A  L  T
ggcgttaaatacgcagtatatggccgtgaggaaggtgaacgcaacaagacaaggcatctt
 G  V  K  Y  A  V  Y  G  R  E  E  G  E  R  N  K  T  S  H  L
caggggtatgtacatttcacgtcaagaaaagaatttggagcgcttaaacgaagccttggg
 Q  G  Y  V  H  F  T  S  S  K  E  F  G  A  L  K  R  S  L  G
gatcgtttccacattgaacagggtaagtagtagacaatgaaatcttgtttatagtatttg
 D  R  F  H  I  E  Q 1564
attatctactgcttatagctagaggcagtgatgcacagaattacaagtactgtactaaa
              1507 A  S  G  S  D  A  Q  N  Y  K  Y  C  T  K
ggaggtgactattgggagactggaagactcgtgggtcaaggacatcgtagcgacttggat
 G  G  D  Y  W  E  T  G  S  L  V  G  Q  G  H  R  S  D  L  D
ggcgtgcttgcaacaattcaatctggggctacttacaatcagatcgccatcgctcatcca
 G  V  L  A  T  I  Q  S  G  A  T  Y  N  Q  I  A  I  A  H  P
caccaatttattagataccacaggggaattagagcatattttggtgcgcttgaacagtta
 H  Q  F  I  S  Y  H  S  G  I  S  A  Y  F  G  A  L  E  Q  L
cgtggacgaagtacgagaacaagatgcttggtattatggggcgattcaggcagcggcaag
 R  G  R  S  T  S  T  S  C  L  V  L  W  G  D  S  G  S  G  K
tcctatacagccagacgagttgcgcgaagctttggaagctgttattacaagacgagagga
 S  Y  T  A  S  R  V  A  R  S  F  G  S  C  Y  Y  K  T  S  G
gaatggtgggacggatacgatgggcaagaagtagttgtaatagatgacttctacggttgg
 E  W  W  D  G  Y  D  G  Q  E  V  V  V  M  D  D  F  Y  G  W
ctaaaatacgatgagttacttaggatattagatgagtatccattacaggttccaattaaa
 L  K  Y  D  E  L  L  S  M  L  D  E  Y  P  L  Q  V  P  I  K
ggcaattttacaaatttccgtagtctattagtcataataaccagtaatgtctgttgggag
 G  N  F  T  N  F  R  S  L  L  V  M  M  T  S  N  V  C  W  E
gagtggtaccatggcacgtggtttaaagaccaagtaaaagcagcacttcaaagaagatta
 E  W  Y  H  G  T  W  F  K  D  Q  V  K  A  A  L  Q  S  S  L
gattggatatataagtacccggaagaaaaggaacaaatgtttacagacaaccatgattta
 D  W  M  Y  K  Y  P  E  E  K  E  Q  M  F  T  D  N  H  D  L
tttatcaataaagaaaattagttaacattatattgtgaaaatttatagtaggtcacttta
 F  I  N  K  E  N ***
------->             846
```

Supplementary material S7 (A and B). The putative Rep encoding open reading frame of canine associated cyclovirus CN9E.

(B) Alignment of the CN9E putative Rep (derived using the invertebrate mitochondrial genetic code (transl_table=5) with alternative initiation codon 'ATA') and the putative CN9E polypeptide (determined using the standard genetic code (transl_table=1) with alternative initiation codon 'TTG)'. The CN9E polypeptide lacked the amino terminal region of Rep including the presence of the conserved rolling cycle replication motifs I and II. Using the standard genetic code with 'ATG' as the initiation codon, CN9E lacked a putative Rep encoding ORF. The deduced amino acid sequences derived from the CN9E putative Rep coding sequences (nt 1780-nt 1564 and nt 1507-nt 846, separated by a putative intron) is shown with blue and green, respectively. The conserved rolling cycle replication and superfamily 3 helicase motifs are underlined. A '*' denotes an identical aa residue, whilst '-' indicates absence of an aa residue. Numbers to the right indicate the positions of the aa for respective sequences. The alignment was performed using the ClustalW program (https://clustalw.ddbj.nig.ac.jp, accessed on September 11, 2021).

```
CN9E/Rep                 MRGVRWCFTCNNYTEEDERAVRALTGVKYAVYGREEGERNKTSHLQGYVHFTSSKEFGAL 60
CN9E/Polypeptide         ------------------------------------------------------------

CN9E/Rep                 KRSLGDRFHIEQASGSDAQNYKYCTKGGDYWETGSLVGQGHRSDLDGVLATIQSGATYNQ 120
CN9E/Polypeptide         -MFIVFDYLLLIARGSDAQNYKYCTKGGDYWETGRLVGQGHRSDLDGVLATIQSGATYNQ 59
                                     * ******************** *************************

CN9E/Rep                 IAIAHPHQFISYHSGISAYFGALEQLRGRSTSTSCLVLWGDSGSGKSYTASRVARSFGSC 180
CN9E/Polypeptide         IAIAHPHQFIRYHRGIRAYFGALEQLRGRSTRTRCLVLWGDSGSGKSYTARRVARSFGSC 119
                         ********** ** ** ************** * **************** *********

CN9E/Rep                 YYKTSGEWWDGYDGQEVVVMDDFYGWLKYDELLSMLDEYPLQVPIKGNFTNFRSLLVMMT 240
CN9E/Polypeptide         YYKTRGEWWDGYDGQEVVVIDDFYGWLKYDELLRILDEYPLQVPIKGNFTNFRSLLVIIT 179
                         **** ************** *************  **********************  *

CN9E/Rep                 SNVCWEEWYHGTWFKDQVKAALQSSLDWMYKYPEEKEQMFTDNHDLFINKEN 292
CN9E/Polypeptide         SNVCWEEWYHGTWFKDQVKAALQRRLDWIYKYPEEKEQMFTDNHDLFINKEN 231
                         ***********************  *** ***********************
```
