# Supplementary material for: Novel Cyclovirus Species in Dogs with Hemorrhagic Gastroenteritis
Source: Viruses. 2021 Oct 26;13(11):2155. doi: 10.3390/v13112155 (PMC8622408; doi:10.3390/v13112155)
Supplement: Supplementary file 1 [file viruses-13-02155-s001.zip › Supplementary figure S8.pdf]

Supplementary figure S8. Alignment of the CN9E putative deduced capsid (Cp) amino acid (aa) sequences derived using the standard genetic code (shown as CN9E/Cp/STD_code) with 'ATG' as initiation codon and invertebrate mitochondrial genetic code (shown as CN9E/Cp/INV-Mit_code) with 'ATG' as the initiation codon. A '*' denotes an identical aa residue. Numbers to the right indicate the positions of the aa for respective sequences.

```
CN9E/Cp/STD_code         MRRRIRRFRRRYRLRTQRRYRKRVKTSSLVRHHRALSSTDNLTVYLTARVRLNLPEPNPS 60
CN9E/Cp/INV-Mit_code     MRRRISRFRRRYRLRTQSRYRKRVKTSSLVRHHRALSSTDNLTVYLTARVRLNLPEPNPS 60
                         ***** *********** ******************************************

CN9E/Cp/STD_code         TPFGYSGKYDLSFFQNEAYTETNLPAVHRFFQYYRIHWIKEELKPAFNVIKNIDPYSYLN 120
CN9E/Cp/INV-Mit_code     TPFGYSGKYDLSFFQNEAYTETNLPAVHSFFQYYRIHWMKEELKPAFNVIKNIDPYSYLN 120
                         **************************** ********* *********************

CN9E/Cp/STD_code         SQTEQLTTWRNRNMTSYVCAPWKRQNPPANELELSITKGAKKIRAHQECRRKFKPAVYTA 180
CN9E/Cp/INV-Mit_code     SQTEQLTTWRNRNMTSYVCAPWKRQNPPANELELSMTKGAKKMRAHQECRRKFKPAVYTA 180
                         *********************************** ****** *****************

CN9E/Cp/STD_code         QQSNTTQQYTFTDMKYSPKIASDANGVRVPHWCSYWFLPYFQITPTNPGTPETTDYPLFE 240
CN9E/Cp/INV-Mit_code     QQSNTTQQYTFTDMKYSPKMASDANGVRVPHWCSYWFLPYFQITPTNPGTPETTDYPLFE 240
                         ******************* ****************************************

CN9E/Cp/STD_code         MWATAKVTYYKFSQYNVN 258
CN9E/Cp/INV-Mit_code     MWATAKVTYYKFSQYNVN 258
                         ******************
```
