# Supplementary material for: Novel Cyclovirus Species in Dogs with Hemorrhagic Gastroenteritis
Source: Viruses. 2021 Oct 26;13(11):2155. doi: 10.3390/v13112155 (PMC8622408; doi:10.3390/v13112155)
Supplement: Supplementary file 1 [file viruses-13-02155-s001.zip › Supplementary table S1.pdf]

Supplementary table S1. Additional primers employed in inverse nested PCRs to amplify the complete genomes of canine associated cycloviruses.

**Canine associated cyclovirus**	**Primer name**	**Primer sequence (5'-3')**	**Primer position**
**CN9E**	CN9E-F1	AGATACCACAGGGGAATTAGAGC [1]	nt 493-nt 515 [2]
	CN9E-R1	GATGAGCGATGGCGATCTGATTG [1]	nt 478-nt 456 [2]
	CN9E-Nested PCR-F2	GCGCTTGAACAGTTACGTGGACG [1]	nt 526-nt 548 [2]
	CN9E-Nested PCR-R2	ATCCAAGTCGCTACGATGTCCTTG [1]	nt 420-nt 397 [2]
**CN16E and CN34**	CN16E-F1	ACGTAATGACCTTGACGACGTTG [3]	nt 339-nt 361 [4]
	CN16E-R1	CTTGTTGTTGTGGTTCCCCATATTC [3]	nt 334- nt 310 [4]
	CN16E-Nested PCR-F2	TTATGCAGTGGGAACTGGGACG [3]	nt 461-nt 482 [4]
	CN16E-Nested PCR-R2	TGGCTGTTTTCTCCGATGATTGATC [3]	nt 242- nt 208 [4]

The primer sequences were designed manually from the partial *rep* sequence of CN9E [1] and CN16E [3]. The partial *rep* (~400 bp) of the canine associated cycloviruses were amplified by nested PCR assays employing pan-*rep* primers (CV-F1, CV-R1, CV-F2 and CV-R2) as previously described by Li et al. [3].

Nucleotide (nt) position corresponds to that of the reverse complement of complete genome sequence of canine associated circovirus CN9E [2] and CN16E [4]. Following the recommendations of the ICTV classification system [1, 2], the first nt of the nonanucleotide motif was considered as 'position one' of the 'CN' sequences.
